# Targeting Skin Aging Hallmarks In Vitro: Antioxidant, Anti-Inflammatory, and Anti-Senescence Effects of Phenolic-Rich Extracts from *Cistus* L. Species

**DOI:** 10.3390/antiox15010149

**Published:** 2026-01-22

**Authors:** Mário Pedro Marques, Euclides Landim, Carla Varela, Ricardo M. F. da Costa, Joana Marques, Luís A. E. Batista de Carvalho, Ana Silva, Maria Teresa Cruz, Rebeca André, Patrícia Rijo, Maria Inês Dias, Aida Carvalho, Paulo J. Oliveira, Célia Cabral

**Affiliations:** 1 Coimbra Institute for Clinical and Biomedical Research (iCBR), Clinic Academic Center of Coimbra (CACC), Faculty of Medicine, University of Coimbra, 3000-548 Coimbra, Portugal; silvamarques@student.uc.pt (M.P.M.); euclides.landim@student.uc.pt (E.L.); 2 Center for Innovative Biomedicine and Biotechnology (CIBB), University of Coimbra, 3000-548 Coimbra, Portugal; anacrs@cnc.uc.pt (A.S.); trosete@ff.uc.pt (M.T.C.); pauloliv@cnc.uc.pt (P.J.O.); 3 Chemical Engineering and Renewable Resources for Sustainability (CERES), Faculty of Sciences and Technology, University of Coimbra, 3030-790 Coimbra, Portugal; carlalvarela@gmail.com; 4 Laboratório Associado para a Química Verde/Rede de Química e Tecnologia (LAQV/REQUIMTE), Molecular Physical-Chemistry R&D Unit, Department of Chemistry, University of Coimbra, 3004-535 Coimbra, Portugal; rmfdcosta@uc.pt (R.M.F.d.C.); marques.jt@uc.pt (J.M.); labc@ci.uc.pt (L.A.E.B.d.C.); 5 Centre for Functional Ecology—Science for People and the Planet (CFE), Department of Life Sciences, University of Coimbra, 3000-456 Coimbra, Portugal; 6 Center for Neuroscience and Cell Biology (CNC-UC), University of Coimbra, 3004-504 Coimbra, Portugal; 7 Faculty of Pharmacy, University of Coimbra, 3000-548 Coimbra, Portugal; 8 CBIOS—Universidade Lusófona’s Research Center for Biosciences & Health Technologies, 1749-024 Lisbon, Portugal; rebeca.andre@ulusofona.pt (R.A.); patricia.rijo@ulusofona.pt (P.R.); 9 Centro de Química Estrutural, Institute of Molecular Sciences, Universidade de Lisboa, Campo Grande, 1749-016 Lisboa, Portugal; 10 Instituto de Investigação do Medicamento (iMed.ULisboa), Faculty of Pharmacy, University of Lisbon, 1649-003 Lisboa, Portugal; 11 Centro de Investigação de Montanha (CIMO), Laboratório Associado para a Sustentabilidade e Tecnologia em Regiões de Montanha (LA SusTEC), Instituto Politécnico de Bragança (IPB), Campus de Santa Apolónia, 5300-253 Bragança, Portugal; maria.ines@ipb.pt; 12 Instituto Politécnico de Bragança (IPB), Campus de Santa Apolónia, 5300-253 Bragança, Portugal; acarvalho@ipb.pt; 13 Centre for Tourism Research, Development and Innovation (CiTUR), Pólo Guarda, Av. Dr. Francisco Sá Carneiro 50, 6300-559 Guarda, Portugal; 14 Fundação Côa Parque, Rua do Museu, 5150-620 Vila Nova de Foz Côa, Portugal; 15 Instituto de Histologia e Embriologia, Faculty of Medicine, University of Coimbra, Rua Larga, Edifício da FMUC, Pólo 1, 2º Piso, 3004-504 Coimbra, Portugal

**Keywords:** *Cistus* spp., plant extract, phenolic compounds, cytoprotective, cytotoxicity, anti-inflammatory, antioxidant, anti-senescence, irritant effects

## Abstract

Plant-based extracts are rich sources of phenolic compounds, which may act as skin antiaging mediators. Herein, *Cistus albidus* L. (Ca), *Cistus ladanifer* L. subsp. *ladanifer* (Cl) and *Cistus salviifolius* L. (Cs) were selected to test whether their phytochemical profile and bioactive potential align to target human skin aging. Hydroethanolic extracts (HEs) were prepared and characterized using infrared vibrational spectroscopy (FTIR-ATR) and liquid chromatography–mass spectrometry (LC-MS). Non-toxic concentrations were screened, and cytoprotective and antioxidant effects were studied in *tert*-butyl hydroperoxide-stimulated normal human dermal fibroblasts (NHDFs). Lipopolysaccharide-stimulated RAW 264.7 macrophages were used to assess anti-inflammatory activity, the Organization for Economic Co-operation and Development (OECD) Test Guideline No. 439 was used to assess irritant effects, and the anti-senescence potential was assessed in etoposide-stimulated NHDFs. A series of enzymatic inhibition assays was performed. All extracts comprised ellagic acid derivatives, as well as myricetin and quercetin derivatives in Cs and Ca. The HE of Cs was also markedly composed of ligstroside. At non-toxic concentrations, cytoprotective effects were observed in NHDFs. However, only Cs and Cl exhibited significant antioxidant activity in these cells (*p* < 0.001 and *p* < 0.0001, respectively). In addition to that, Cl demonstrated highly significant anti-inflammatory (*p* < 0.0001) and anti-senescence (*p* < 0.0001) effects. Cs and Cl showed a remarkable potential to inhibit elastase; in addition, Cs also showed anti-hyaluronidase and anti-tyrosinase activities. Meaningfully, Cs and Cl extracts did not exhibit skin irritant effects. The unveiled potential of Cl in skin aging offset highlights the need to elucidate the detailed mechanisms of action, paving the way for the development of skin anti-aging formulations.

## 1. Introduction

The skin is primarily composed of a three-layered structure, consisting of (1) the epidermis, in which keratinocytes are the main cells-type at various differentiation stages; (2) the dermis, where fibroblasts play a crucial role in synthesizing extracellular matrix, contributing to the preservation of skin structure and elasticity; and lastly, (3) the hypodermis, fundamentally composed of adipocytes. Covering approximately 1.8 m^2^ of the body’s external surface, the skin is the largest human organ, serving as the primary barrier against pathogens, ultraviolet (UV) radiation, and environmental chemicals or pollutants [1,2]. These stressful conditions may trigger higher levels of reactive oxygen species (ROS) such as superoxide anions (O_2_^•−^), hydrogen peroxide (H_2_O_2_), hydroxyl radicals (^•^OH), and reactive nitrogen species (RNS) such as nitric oxide (NO) [3]. An imbalance between pro-oxidant and antioxidant species may contribute to oxidative injury of biomolecules like structural proteins, lipids, and DNA, promoting cellular senescence or even cell death, processes that are often at the core of both skin aging and pathology [3,4]. Nevertheless, the human body possesses physiological and homeostatic levels of oxidative and inflammatory factors, through an endogenous human antioxidant system constituted by enzymatic and non-enzymatic antioxidants which minimize the oxidative damage in the skin. Cellular antioxidant mechanisms include enzymatic components, as well as non-enzymatic defenses, which are mainly acquired through the diet. These include vitamins (e.g., α-tocopherol and vitamin C), minerals and cofactors, sulfur-containing compounds, non-protein nitrogenous substances, and plant-derived metabolites [4,5,6]. From this perspective, plants constitute a valuable source of chemopreventive compounds, with particular emphasis on polyphenols. These molecules stand out due to their capacity to scavenge ROS/RNS and modulate signaling pathways related to oxidative stress. Their hydroxyl groups are readily oxidized to quinones, endowing them with a powerful redox buffering capacity [6].

The Mediterranean basin is considered a global hotspot of biodiversity [7] and plants native to this biogeographic region are subjected to harsh edaphoclimatic conditions such as high temperatures, prolonged drought, intense UV radiation, and frequent wildfires, which are typical of this biome. These demanding conditions induced plants to develop protective metabolic strategies, particularly the accumulation of phenolic compounds which, for example, confer protection from photo-oxidative stress conditions [8]. Plants from the genus *Cistus* L. are well represented in the Mediterranean-influenced climatic zone and are known to produce ellagitannins, flavonoids, and phenolic acids derivatives [9]. Interestingly, several works have pointed the beneficial effects of such compounds on human skin cells, constituting natural-based solutions to counteract skin aging [10].

Recently, an emerging interest has driven research studies on *Cistus* plants [7], particularly those regarding skin application [8,11,12]. Furthermore, ethnomedical practices highlight the use of the *Cistus* species as traditional remedies for microbial infections, with particular emphasis on their application in the treatment of external wounds [7,13]. To build on this knowledge, we have selected three *Cistus* species that are particularly abundant in the hillsides of a Portuguese Northeastern region of Portugal, named Côa Valley, which are *Cistus albidus* L. (Ca), *Cistus ladanifer* L. subsp. *ladanifer* (Cl), and *Cistus salviifolius* L. (Cs). Important ethnobotanical studies provide evidence of their use in the treatment of several skin conditions in different territories of the Iberian Peninsula. As such, *C. albidus*, locally called *hierba lobera* in the South Alava region (Spain), has traditional applications in the treatment of skin injuries and infections resulting from wolf bites, with decoctions of its aerial parts used as external washes [14]. Also, in the region of Biscay and Alava (Spain), the leaves from *C. salviifolius* have ancient topical applications for the treatment of topical wounds and cuts, dog bites, and external ulcers [15]. For instance, in Trás-os-Montes (northern Portugal), the flowers and leaves of *C. ladanifer* are externally used in cataplasms and baths as an anti-infectious and wound healing treatment [16].

Considering this, although few studies have described the phytochemical composition or selected bioactivities of the *Cistus* species, most have examined isolated endpoints and lack an integrated evaluation relevant to the context of skin aging. Our work aims to advance the current state of the art regarding these plants species by combining detailed chemical profiling with a broad panel of biological assays that simultaneously address key hallmarks of skin aging, including oxidative stress, inflammation, senescence, enzyme-mediated extracellular matrix components degradation, and robust cell toxicity endpoints. Furthermore, the direct comparison of three species of the same botanical genus under standardized conditions, together with validated safety assessments, offers new insights into species-specific activities and their translational relevance for anti-aging applications. Overall, this multidimensional approach provides a more comprehensive understanding than previously reported of the potential topical application of *Cistus* extracts as promising candidates to be included in innovative anti-aging formulations.

## 2. Materials and Methods

### 2.1. Reagents and Chemicals

From Sigma-Aldrich (St. Louis, MO, USA) we obtained sodium bicarbonate (S5761), D-(+)-Glucose (G8270), Dulbecco’s Modified Eagle’s Medium without glucose (DMEM5030), L-glutamine (G3126), sodium pyruvate (P5280), penicillin–streptomycin solution (P4333), 0.25% Trypsin-EDTA solution (T4049), trypan blue solution (T8154), dimethyl sulfoxide (DMSO) (D2438), 2,2-diphenyl-1-picrylhydrazyl (D9132), 2,4,6-tris(2-pyridyl)-s-triazine (≥99%) (93285), ammonium acetate (≥98%) (A7262), butylated hydroxytoluene (BHT) (≥99%) (W218405), gallic acid (97.5–102.5% (titration)) (G7384), (+)-Catechin hydrate (≥98%) (C125), ferulic acid (PHR1791), quercetin (≥95%) (Q4951), neocuproine (≥98%) (N1501), sodium acetate (≥99%) (S5636), trolox (97%) (238813), iron (III) chloride FeCl_3_·6(H_2_O), (≥98%) (31232-M), Lipopolysaccharide (LPS) from Escherichia coli serotype 026:B6 (L2654), acetylcholinesterase from Electrophorus electricus (electric eel) (C3389), acetylthiocholine iodide (≥98% (TLC)) (A5751), 5,5′-Dithio-bis-(2-nitrobenzoic Acid) (DTNB) (322123), N-(1-naphthyl)-ethylenediamine dihydrochloride (>98%) (222488), sulphanilamide (≥99%) (S9251), tert-Butyl hydroperoxide solution (t-BHP) 70 wt. % in H2O (458139), hydrogen peroxide (H_2_O_2_) 30% (*w*/*w*) in H_2_O (H1009), thiazolyl blue tetrazolium bromide (MTT) (≥97.5%) (M5655), sodium dodecyl sulfate (SDS) (≥98.5%) (L4509), N-Succinyl-Ala-Ala-Ala-p-nitroanilide (≥98%) (S4760), tyrosinase from mushroom (T3824), L-Tyrosine (99.0–101.0%) (T8566), kojic acid (≥98.5%) (K3125), and sodium hydroxide (NaOH) (≥98%) (S5881). The reagents aluminum chloride (AlCl_3_, for synthesis) (801081), copper (II) chloride (CuCl_2_, for synthesis) (818247), potassium chloride (KCl) (≥99%) (104936), sodium carbonate (≥99.5%) (106392), and sodium acetate trihydrate (CH_3_COONa^•^3H2O) (99.5%) were obtained from Merck (Oeiras, Portugal). Fetal bovine serum (FBS) (S1810-500) and DMEM Low glucose (P0061-N10L) were acquired from BioWest (Nuaillé, France). Resazurin sodium salt (B21187), Sulforhodamine B sodium salt (SRB) (A14769.06), Tris Base (BP152-1), 2,2′-Azino-bis(3-ethylbenzothiazoline-6-sulphonic acid) diammoniumsalt (≥98%) (J65535.06), *p*-Coumaric acid (*trans*-4-Hydroxycinnamic acid) (≥97.5%) (A15167.14), 2′,7′-dichlorodihydrofluorescein diacetate (H_2_DCFDA) (D399), phosphoric acid 85% aqueous solution (A18067.0D), and potassium metaborate hydrate (KBO_2_^•^H_2_O) (99.98%) (047365.30) were purchased from Thermo Scientific Chemicals (Waltham, MA, USA). Glacial acetic acid (27225), methanol (24229), ethanol absolute (24194), hydrochloric acid (HCl) (37–38.0%) (30721), and potassium persulfate (≥99%) (216224) were bought from Honeywell (Carnaxide, Portugal). The Folin–Ciocalteu’s reagent (251567.1609) was purchased from Panreac (Barcelona, Spain). From Carlo Erba Reagents (Milan, Italy) we acquired calcium chloride dihydrate (CaCl_2_^•^2H_2_O) (99.0–105.0%) (327607). Kaempferol (≥98.0%) (7W-GP7425), epicatechin (≥95.0%) (BP-BP0538), and myricetin (≥97.0%) (3B-M2131) were bought from Cymit Química (Pamplona, Spain), while from TargetMol (Linz, Austria) (−)-epigallocatechin gallate (EGCG) (99.43%) (T2988) was purchased.

### 2.2. Plant Material

Harvested plant material followed a botanical identification carried out by Rosa Pinho, Herbarium Curator of the University of Aveiro, and voucher specimens were deposited at the Herbarium (AVE) of the same university. Detailed information about the harvesting of plants is presented in the Supplementary Materials (Table S1).

### 2.3. Hydroethanolic Extracts Preparation

The HEs were prepared by the procedure previously described by Amorim and collaborators [17]. Further information about the preparation of extracts is presented in the Supplementary Materials (Table S1).

### 2.4. FTIR-ATR Spectroscopy Analysis

Fourier-transformed mid-infrared (FTIR) spectra were acquired in attenuated total reflectance (ATR) mode for the obtained freeze-dried extracts in the 3700–400 cm^−1^ range, following the approach previously by Marques and collaborators [18]. Preliminarily, spectra from standard phenolic compounds (Figure S1, Supplementary Materials) were acquired in the same conditions as for plant extracts, as these are among the most common constituents in plant-derived samples.

### 2.5. HPLC–DAD–ESI/MSn Analysis

The extracts were analyzed following the procedure described by Bessada and collaborators [19] using a Dionex Ultimate 3000 UPLC (Thermo Scientific, San Jose, CA, USA) system equipped with a diode array detector coupled to an electrospray ionization mass detector (HPLC-DAD-ESI/MSn).

### 2.6. Major Phenolics Estimation and Cell-Free Antioxidant Activity Assessment

Spectrophotometric quantifications of total phenolic content (TPC) and total flavonoid content (TFC), and cell-free antioxidant assays were performed as previously described by Marques and collaborators [20]. For these assays, three independent experiments were performed in triplicate.

### 2.7. Cell Culture

Normal human dermal fibroblasts (NHDFs, CC-2511, Lonza AG Group, Basel, Switzerland) were cultured in low-glucose (5 mM) Dulbecco’s modified Eagle’s medium (DMEM) (pH 7.3), The human keratinocytes (HaCaT, CLS 300493, Eppelheim, Germany) were cultured in DMEM high glucose (25 mM) (pH 7.3). The media of both NHDF and HaCaT cell lines were supplemented with 10% (*v*/*v*) heat-inactivated FBS and 1% (*v*/*v*) penicillin–streptomycin solution. The mouse leukemic macrophage cell line (RAW 264.7, ATCC TIB-71, Manassas, VA, USA) was cultured in DMEM high glucose (25 mM) (pH 7.3), supplemented with 10% (*v*/*v*) non-inactivated FBS, 1% (*v*/*v*) penicillin–streptomycin solution.

### 2.8. Cell Metabolic Activity

The effect of HEs on the metabolic activity of normal skin cells, specifically NHDFs and HaCaT, was evaluated following the resazurin reduction principle and the protocol established by Silva and collaborators [21].

### 2.9. Cellular Protein Content

Sulforhodamine B (SRB) assay was performed to evaluate the influence of extracts on cellular protein content as previously stated by Silva and collaborators [21].

### 2.10. Cell-Free Enzymatic Inhibition Assays

The anti-elastase and anti-tyrosinase assays were assessed following the procedures of Andrade and collaborators [22] and the anti-hyaluronidase as stated by Ratnasooriya et al. [23]. For these assays, five independent experiments were carried out in triplicate. The results were expressed as a percentage (%) of inhibition.

### 2.11. Measurement of Cellular Nitrites Production

The procedure previously published by Moreira and collaborators [24] was carried out to determine nitrite production in lipopolysaccharide-stimulated RAW 264.7 cells.

### 2.12. Evaluation of Cytoprotective Efficiency

The cytoprotective effect of HEs in the presence of the oxidative stressors *tert*-butyl hydroperoxide (*t*-BHP) and hydrogen peroxide (H_2_O_2_) was evaluated in NHDFs following previous procedures [25]. Different peroxides were chosen based on their different mechanisms of action that differ slightly, and preliminarily, dose–response curves were produced to find a suitable concentration of *t*-BHP (0.5 mM) and H_2_O_2_ (1.5 mM).

### 2.13. Determination of Intracellular Oxidative Stress

To evaluate intracellular oxidative stress, the oxidation of the H_2_DCFDA fluorescent dye was measured as previously described by Pinho and collaborators [26].

### 2.14. Skin Irritant Effects

Skin irritation was evaluated through a standard operating procedure using the SkinEthic™ Reconstructed Human Epidermis (RHE) model (EPISKIN Laboratories, Lyon, France), in compliance with OECD guidelines, and as previously described [24].

### 2.15. Senescence-Associated β-Galactosidase Activity

The anti-senescence potential was assessed as previously stated [24] with minor changes. Briefly, NHDF cells were seeded in 12-well plates at 1.5 × 10^4^ cells/well and allowed to proliferate for 24 h. Afterwards, *C. ladanifer* HE was added to the cells for another 24 h. Then, the HE was removed, and etoposide (12.5 μM) was added for 24 h more. After inducing senescence, fresh medium without HE and/or etoposide was added, and the cells were allowed to develop a senescence-associated phenotype for 6 days. The senescence-associated β-galactosidase activity of etoposide-stimulated NHDFs was evaluated following the manufacturer’s instructions for the commercial kit used (CS0030; Sigma-Aldrich). Senescent cells were ultimately imaged with a widefield microscope at 40× magnification by counting the proportion of X-gal positive cells across four randomly chosen fields. Five independent experiments, with two experimental replicates per condition, were performed.

### 2.16. Statistical Analysis

The results are presented as the mean ± standard deviation (SD) of the indicated number of independent experiments. D’Agostino’s, Pearson’s, and Shapiro–Wilk’s normality tests were conducted to check the normality of the data distribution. All calculations for descriptive statistics, one-way analysis of variance (ANOVA), as well as Tukey’s, Dunnett’s, and Sidak’s range tests were performed using GraphPad Prism 9.0 software (GraphPad Software, La Jolla, CA, USA) considering the following significance values: * *p* < 0.05, ** *p* < 0.01, *** *p* < 0.001, **** *p* < 0.0001, as well as ^#^ *p* < 0.05, ^##^ *p* < 0.01, ^###^ *p* < 0.001, ^####^ *p* < 0.0001 for Dunnett’s and Sidak’s tests, while for Tukey’s test significant values (*p* < 0.05) were represented by lowercase letters (a–d).

## 3. Results

### 3.1. FTIR-ATR Spectroscopy

**Figure 1:**
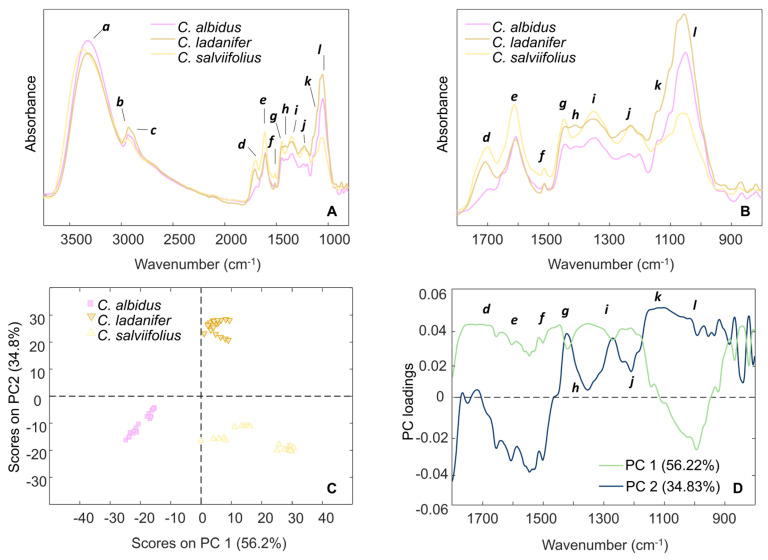
(**A**) Mean FTIR-ATR spectra in the range 3700–800 cm^−1^; (**B**) mean FTIR-ATR spectra in the region 1800–800 cm^−1^; (**C**) plot of principal component one (PC1) and principal component two (PC2) scores; and (**D**) PC loadings plot of *C. albidus*, *C. ladanifer,* and *C. salviifolius* HEs. Relevant spectral range assignments are summarized in Table 1.

As an initial exploratory step, the FTIR-ATR spectra of the hydroethanolic extracts (HEs) from the studied *Cistus* species were recorded in the range 3700–800 cm^−1^ (Figure 1A), as well as from standard phenolic compounds (Figure S1). Spectral bands were assigned according to chemical functional group (Table 1). Notably, the region 1800–800 cm^−1^ displayed pronounced differences among the samples, revealing substantial compositional diversity (Figure 1B).

To explore inter-sample variability, principal component analysis (PCA) was applied to the FTIR-ATR data. The first two principal components (PC1 and PC2) accounted for 91.0% of the total variance, with PC1 explaining 56.2% and PC2 34.8% (Figure 1C). Along PC1, the spectra of the HE from *C. albidus* flowering aerial parts was clearly separated, positioned on the negative side. Conversely, the spectra of *C*. *ladanifer* and *C*. *salviifolius* HEs clustered on the positive side of PC1. This pattern indicates a higher degree of compositional similarity between *C. ladanifer* and *C. salviifolius* compared with *C. albidus*. Further separation was observed along PC2, with *C*. *ladanifer* extracts positioned in the positive quadrant, indicating unique spectral characteristics compared to both *C*. *albidus* and *C*. *salviifolius*. The interpretation of the corresponding loading plots (Figure 1D) highlights the key spectral regions responsible for this segregation, providing further insight into the relative compositional differences between the HEs of the studied *Cistus* species.

### 3.2. Total Phenolic and Total Flavonoid Contents and Cell-Free Antioxidant Activity

**Figure 2:**
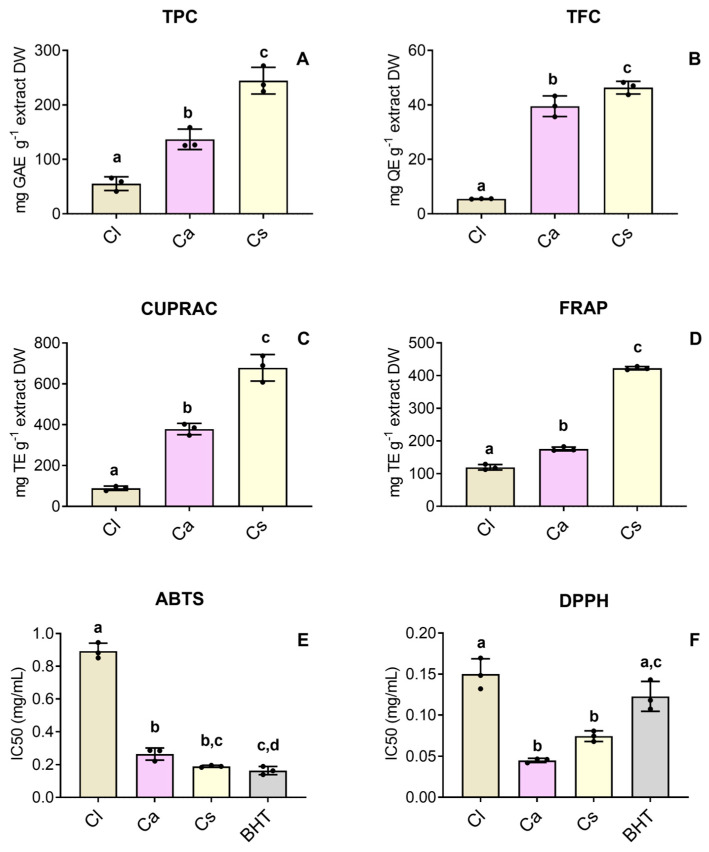
(**A**) Total phenolic content (TPC) (mg GAE g^−1^ extract DW); (**B**) total flavonoid content (TFC) (mg QE g^−1^ extract DW); (**C**) cupric (CUPRAC) and (**D**) ferric (FRAP) reducing powers (mg TE g^−1^ extract DW). Free radical scavenging activity (**E**) ABTS and (**F**) DPPH (IC_50_ values, mg/mL of HE) using BHT (butylated hydroxytoluene) as positive control. Different assays are represented in differently scaled graphs. Panels are labeled with uppercase letters (**A**–**D**) to identify each assay. Bars represent the mean ± SD of three independent experiments performed in triplicate. Different lowercase letters (a–d) indicate significant differences between groups. The statistical analysis was carried out by one-way ANOVA, followed by Tukey’s post hoc test (*p* < 0.05). Abbreviations: Ca, *C. albidus*; Cl, *C. ladanifer*; and Cs, *C. salviifolius*.

Regarding TPC (Figure 2A) and TFC (Figure 2B) determinations, the ranking order points consistently to greater amounts of both TPC and TFC in *C. salviifolius*, followed by *C. albidus,* and lastly, *C. ladanifer*. Similarly, the studied HEs registered the same tendency regarding the CUPRAC and FRAP assays, being more effective in reducing copper than iron (Figure 2C and Figure 2D, respectively), with substantially higher CUPRAC activity, ranging between 89.102 ± 10.88 TE g^−1^ extract DW in *C. ladanifer* and 678.517 ± 65.08 TE g^−1^ extract DW in *C. salviifolius*. Also, the HEs of *C. salviifolius* and *C. albidus* presented a strong ability to inhibit DPPH^•^ (Figure 2F), even outperforming the reference antioxidant butylated hydroxytoluene (BHT; IC_50_ = 0.123 ± 0.018 mg/mL). However, regarding the ABTS assay, the studied HEs did not show the same potential, and only *C. salviifolius* became close to the BHT inhibition potential (Figure 2E). The determined values (mean ± SD) in these assays are summarized in the Supplementary Materials (Table S2).

### 3.3. HPLC-DAD-ESI/MSn Analysis

**Figure 3:**
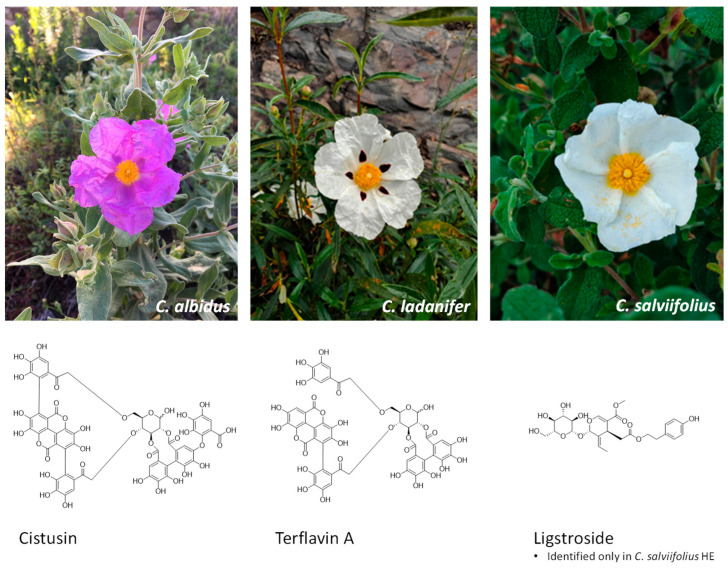
From left to right, the flowering aerial parts of *C. albidus*, *C. ladanifer*, and *C. salviifolius*, harvested in Côa Valley (Portugal), and the chemical structures of the major phenolic compounds identified in the studied extracts. Plants’ photographs were captured by Mário Pedro Marques. ChemDraw Software v.14.0 was used to draw the chemical structures.

Among the studied samples, 42 compounds were tentatively identified, including 18 in the HE from *C. albidus*, 9 in the HE from *C. ladanifer* and 15 in the *C. salviifolius* HE. The chromatographic and mass responses, as well as the respective tentative identification of the phenolic compounds found, are described in Table 2. The chemical structures of the most relevant phenolics identified in HEs are illustrated in Figure 3. The illustrative phenolic profiles recorded at 280 and 370 nm, are presented in the Supplementary Materials (Figure S2).

Similarly to *C. ladanifer* and *C. salviifolius*, two ellagic acid derivatives were found in *C. albidus* HE: peak 2*^Ca^* (terflavin A) and peak 4*^Ca^*(cistusin). Phenolic acids were mainly represented by peak 3*^Ca^* ([M-H]^−^ *m*/*z* 337, 3-*O*-*p*-coumaroylquinic acid) and vanillic acid hexoside (18*^Ca^*, [M-H]^−^ *m*/*z* 329). Peak 1*^Ca^* ([M-H]^−^ *m*/*z* 305, gallocatechin) and peak 5*^Ca^* comprised the flavan-3-ols group. Major phenolics were *O*-glycosylated flavonoids: kaempferol 7-*O*-(6″-*p*-coumaroyl)hexoside (16*^Ca^*), myricetin, and quercetin derivatives linked to hexosyl, malonyl-hexosyl, pentosyl, and deoxyhexosyl moieties, represented by the peaks 6*^Ca^* to 15*^Ca^*. The *C. ladanifer* HE showed *C*- and *O*-glycosylated flavonoids (peaks 5′*^Cl^*, 5⁗*^Cl^*, 6′*^Cl^*, 18′*^Cl^*), ellagitannins (terflavin A 2*^Cl^*, cistusin 4*^Cl^*), and dihydroxy-tetramethoxyflavone (5″*^Cl^*). *C. salviifolius* HE presented ellagic acid derivatives (terflavin A 1*^Cs^*, cistusin 2*^Cs^*), and a secoiridoid (3*^Cs^*, [M-H]^−^ *m*/*z* 453), and several flavonoids were found, including various myricetin and quercetin derivatives (peaks 8*^Cs^*–13*^Cs^*), confirming that plants from the genus *Cistus* have a highly diverse phenolic profile.

### 3.4. Cytotoxic Effects on Normal Human Skin Cells

**Figure 4:**
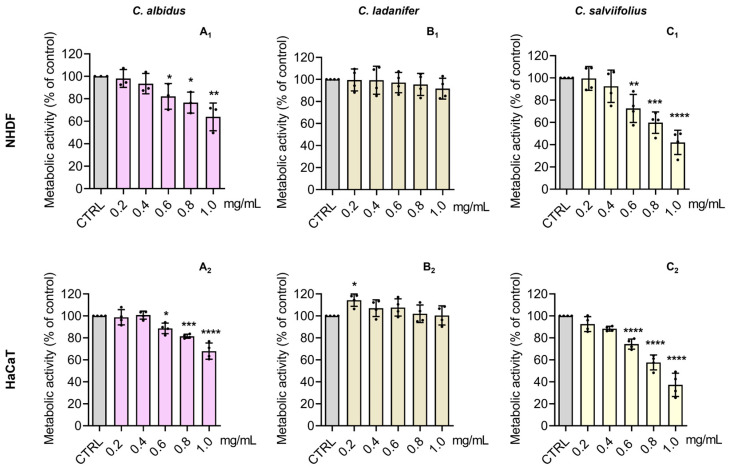
Effect of the HE of *C. albidus* (**A_1_**,**A_2_**), *C. ladanifer* (**B_1_**,**B_2_**), and *C. salviifolius* (**C_1_**,**C_2_**) on the metabolic activity of NHDF and HaCaT cells, respectively. Cells were treated with HE (0.2–1.0 mg/mL) for 24 h and then the effect on metabolic activity was evaluated through the Alamar blue^®^ assay. Untreated cells were used as the control (CTRL). Results are expressed as a percentage (%) of metabolic activity relative to the CTRL and represent the mean ± SD of four independent experiments, each one performed in triplicate. The statistical analysis was carried out by one-way ANOVA followed by Dunnett’s multiple comparison test (* *p* < 0.05, ** *p* < 0.01, *** *p* < 0.001, and **** *p* < 0.0001 versus CTRL).

**Figure 5:**
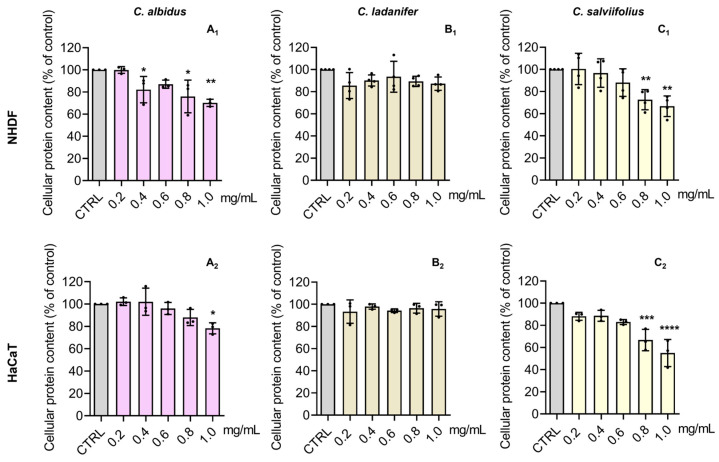
Effect of the HE of *C. albidus* (**A_1_**,**A_2_**), *C. ladanifer* (**B_1_**,**B_2_**), and *C. salviifolius* (**C_1_**,**C_2_**) on the cellular protein content of NHDF and HaCaT cells, respectively. Cells were treated with HE (0.2–1.0 mg/mL) for 24 h and then the effect on cellular protein content was evaluated through the SRB assay. Untreated cells were used as the control (CTRL). Results are expressed as a percentage (%) of cellular protein content relative to the CTRL and represent the mean ± SD of four independent experiments, each one performed in triplicate. The statistical analysis was carried out by one-way ANOVA followed by Dunnett’s multiple comparison test (* *p* < 0.05, ** *p* < 0.01, *** *p* < 0.001, and **** *p* < 0.0001 versus CTRL).

NHDF cells have been used as a toxicological model for skin applications [25,43] and skin fibroblasts are often employed to assess plant-derived phenolics [10]. HaCaT cells, resembling in vivo keratinocytes, are also suitable for cytotoxicity studies [24]. The cytotoxic effect of HEs (0.2–1.0 mg/mL) from *Cistus* spp. was tested in NHDF and HaCaT cells regarding metabolic activity (Figure 4) and cellular protein content (Figure 5). *C. albidus* and *C. salviifolius* showed no significant impact at ≤0.2 mg/mL and ≤0.4 mg/mL, respectively, while *C. ladanifer* caused no relevant effects across tested doses. Considering both parameters, the toxicity ranking was *C. ladanifer* (≤1.0 mg/mL, least toxic) < *C. salviifolius* (≤0.4 mg/mL) < *C. albidus* (≤0.2 mg/mL, most toxic).

### 3.5. Antioxidant and Cytoprotective Effects

**Figure 6:**
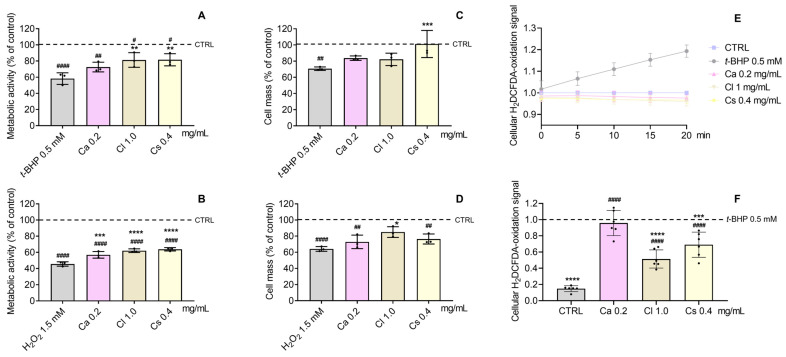
Cytoprotective effect of HE against *t*-BHP (**A**,**C**) and H_2_O_2_-induced (**B**,**D**) metabolic activity and cellular protein content decrease, respectively, in NHDF cells pre-incubated for 24 h with HE, before *t*-BHP (0.5 mM) or H_2_O_2_ (1.5 mM) application for 3 h. (**E**) Monitorization of the pro-oxidant effect of *t*-BHP in comparison to the HE-treated and CTRL NHDF cells. (**F**) Protective effect of HE upon oxidative stress in NHDFs, pre-incubated for 24 h with HE, before *t*-BHP (0.5 mM) application. Untreated cells were used as the control (CTRL). The effects on cell metabolic activity and cellular protein content were evaluated by the Alamar blue^®^ and SRB assays, respectively. Cellular oxidative stress was determined by the H_2_DCFDA assay. Results represent mean ± SD of three (**A**–**D**) and six (**E**,**F**) independent experiments, performed in triplicate. Statistical analysis was performed by one-way ANOVA followed by Dunnett’s multiple comparison test (* *p* < 0.05, ** *p* < 0.01, *** *p* < 0.001, **** *p* < 0.0001 versus *t*-BHP and/or H_2_O_2_) and (^#^ *p* < 0.05, ^##^ *p* < 0.01, ^####^ *p* < 0.0001 versus CTRL). Abbreviations: *t-*BHP, *tert*-butyl hydroperoxide; H_2_O_2_, hydrogen peroxide; H_2_DCFDA, 2′,7′-dichlorodihydrofluorescein diacetate; Ca, *C. albidus*; Cl, *C. ladanifer*; and Cs, *C. salviifolius*.

According to our results (Figure 6), *t*-BHP (0.5 mM) and H_2_O_2_ (1.5 mM) decreased cell metabolic activity by ~40% and ~60%, respectively. *C. salviifolius* and *C. ladanifer* counteracted *t*-BHP effects (*p* < 0.01) (Figure 6A), while all HEs antagonized H_2_O_2_-induced decrease (Figure 6B). For cellular protein content, only *C. salviifolius* (*p* < 0.001 versus *t*-BHP) and *C. ladanifer* (*p* < 0.05 versus H_2_O_2_) prevented reductions (Figure 6C and Figure 6D, respectively). Given these cytoprotective effects, intracellular oxidative stress was assessed via H_2_DCFDA assay (Figure 6E,F). No pro-oxidant activity was observed in HEs-treated NHDFs, unlike *t*-BHP, which significantly increased oxidation (Figure 6E). *C. ladanifer* (*p* < 0.0001) and *C. salviifolius* (*p* < 0.001) markedly reduced oxidative stress in *t*-BHP-treated cells, whereas *C. albidus* showed no protection (Figure 6F).

### 3.6. Anti-Inflammatory Effects Assessed by Nitrite Quantification

**Figure 7:**
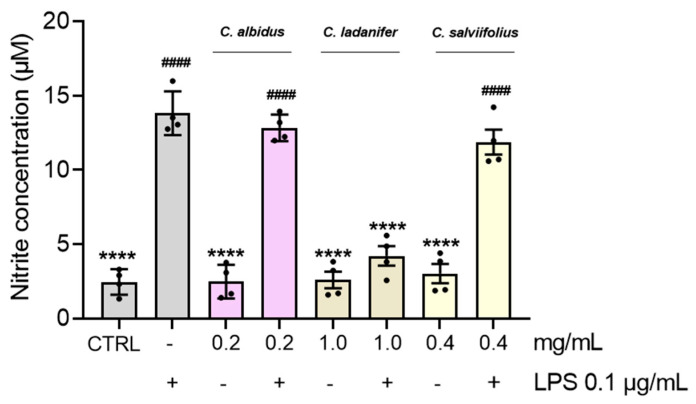
Effect of HEs on LPS-stimulated RAW 264.7 macrophages. The cells were treated with the highest non-toxic concentration of HE, in the absence (−) and presence (+) of 0.1 µg/mL LPS, for 24 h. Untreated cells were used as the control (CTRL). The results were expressed as nitrite concentration (µM) and represent the mean ± SD of four independent experiments, each one performed in triplicate. Statistical analysis was performed by one-way ANOVA followed by Dunnett’s and Šidák’s multiple comparisons tests (**** *p* < 0.0001 versus LPS and ^####^ *p* < 0.0001 versus CTRL).

Nitrite levels were investigated in LPS-activated macrophages to determine the anti-inflammatory potential of HEs at their highest non-toxic concentrations (Figure 7). Therefore, macrophages were subject to HE treatment in the presence (+) of LPS to unveil their potential anti-inflammatory effects, also without (−) LPS stimulation, showing the absence of pro-inflammatory effects on these cells, with a production of nitrites comparable to the CTRL. A cytotoxic assessment of these HEs on RAW 264.7 cells was also carried out (Supplementary Materials, Figure S3). Overall, the results showed a significant inhibition of nitrite production (*p* < 0.0001) when macrophages were treated with *C. ladanifer* HE by reducing nitrite levels by more than half compared with cells treated with LPS alone.

### 3.7. Cell-Free Evaluation of Enzyme Inhibitory Activity

The efficacy of *Cistus* spp. HEs, at their highest non-cytotoxic concentrations, in inhibiting the activity of four key enzymes associated with skin aging was assessed using cell-free enzymatic assays (Table 3). In general, *C. salviifolius* HE consistently presented a remarkable ability to inhibit all tested enzymes, with comparable inhibition rates to the used positive controls. Alongside this, the HE of *C. ladanifer* also showed remarkable potential against elastase inhibition (around 65%), both presenting a comparable effect to the positive control. Concerning hyaluronidase’s inhibition, neither *C. albidus* nor *C. ladanifer* presented the capacity to inhibit this enzyme. Regarding tyrosinase inhibitory activity, *C. salviifolius* reached the effect of the positive control kojic acid.

### 3.8. Skin Irritation

**Figure 8:**
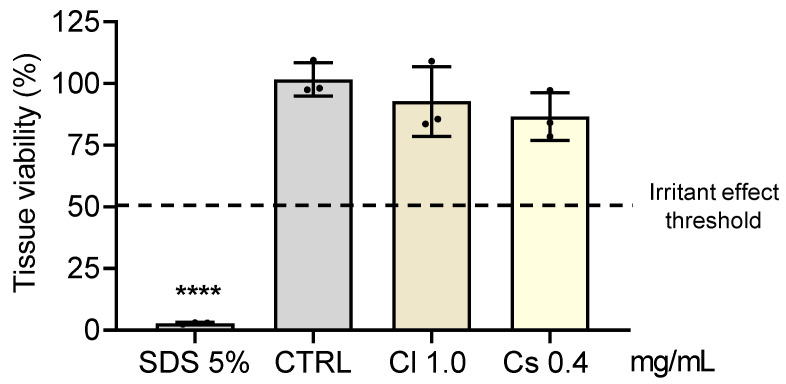
Skin irritation test. HE irritancy was evaluated in a reconstructed human epidermis 3D model (SkinEthic^TM^ RHE). The inserts were treated for 42 min in the absence (control–CTRL) or in the presence of HEs or with 5% (*w*/*v*) sodium dodecyl sulfate (SDS, an irritant used as positive control). The tissue viability was assessed by the MTT assay. Results are the mean ± SD of three independent tissues, and tissue viability is expressed as a % of CTRL (tissue exposed to PBS). The statistical analysis was performed by one-way ANOVA, followed by Dunnett’s multiple comparison test (**** *p* < 0.0001 versus CTRL). Abbreviations: Cl, *C. ladanifer*, and Cs, *C. salviifolius*.

Considering the promising results obtained throughout this study with *C. salviifolius* and *C. ladanifer* extracts, their potential to evoke skin irritation was evaluated in a reconstructed human epidermis 3D model (SkinEthic™ Reconstructed Human Epidermis), in compliance with the OECD Test Guideline No. 439. According to ISO 10993-10: 2010 [44], a substance is considered a non-irritant if it does not reduce tissue viability to ≤50% (as observed for SDS, used as positive control). Since the tissues exposed to both extracts presented tissue viabilities higher than 50%, the results demonstrated the absence of skin-irritating effects for these HEs (Figure 8).

### 3.9. Anti-Senescence Potential

**Figure 9:**
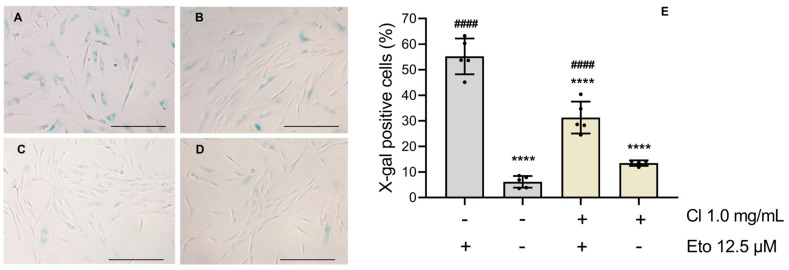
Effect of *C. ladanifer* HE on etoposide-stimulated NHDF cells. Representative bright-field images of NHDF cells (**A**) treated for 24 h with etoposide, (**B**) pre-treated with *C. ladanifer* HE for 24 h before adding etoposide, (**C**) untreated cells used as control (CTRL), (**D**) *C. ladanifer* HE-treated cells without adding etoposide, and determined (**E**) percentages (%) of X-gal positive cells. Etoposide (12.5 µM) was used as a senescence inducer. Scale bar means 500 µm. The results represent the mean ± SD of five independent experiments, each one performed in duplicate. Statistical analysis was performed by one-way ANOVA followed by Dunnett’s and Šidák’s multiple comparisons tests (**** *p* < 0.0001 versus etoposide and ^####^ *p* < 0.0001 versus CTRL). Abbreviations: Eto, etoposide; Cl, *C. ladanifer*.

Considering the potential of *C. ladanifer* HE on several age-related skin dysfunctions tested in this work, its effect on cellular senescence was assessed in terms of senescence-associated β-galactosidase activity (Figure 9). As such, etoposide was shown to evoke a significant (*p* < 0.0001) increase in X-galactose (X-gal)-positive cells (50–60%; Figure 9A,E), in comparison to untreated NHDF cells (Figure 9C). In turn, for the group of pre-treated NHDF cells (Figure 9B) with *C. ladanifer* HE, the number of X-gal-positive cells (around 30%; Figure 9E) was significantly lower (*p* < 0.0001).

## 4. Discussion

In this study, we prospected the potential of three *Cistus* spp. for the development of innovative plant-based solutions to target skin aging, and for that purpose, hydroethanolic extractions (80:20%, *v*/*v*) were employed for their efficiency in recovering a broad range of polar and non-polar compounds [17]. As such, we first established their chemical profiles through FTIR-ATR spectroscopy and the chemometric analysis of the FTIR-ATR spectra revealed distinct clustering patterns, with *C*. *ladanifer* and *C*. *salviifolius* showing a closer compositional similarity to each other than to *C*. *albidus*. This distinction was particularly evident in the region 1800–800 cm^−1^, with notable variation in the intensity of the carbonyl stretching band around 1701–1693 cm^−1^ (*d*), which is prominent in *C*. *salviifolius* and *C*. *ladanifer* spectra, but weak or absent in *C*. *albidus*. This feature corresponds to C=O stretching in carboxylic groups (COOH) [29,30,33], and aligns with the HPLC-PDA-ESI-MS^n^ data, which revealed a relatively higher abundance of ellagic acid derivatives in *C*. *salviifolius* and *C*. *ladanifer*. Furthermore, this relatively higher abundance of ellagic acid derivatives in the HEs of *C*. *salviifolius* and *C*. *ladanifer*, compared with *C*. *albidus*, is also consistent with the more intense FTIR-ATR signals observed at band *d* (*ν*(C=O) in COOH), band *e* (*ν*(C=C)), and band *f* (*ν*(C-C) aromatic). These bands reflect the presence of aromatic ring systems and are consistent with a higher phenolic acid content in *C. salviifolius* when compared with *C. ladanifer* and *C. albidus*.

Additionally, these findings were further supported by the TPC assay based on GAE, where the highest results were observed for this species, as well as the results from the TFC, CUPRAC, and FRAP assays, altogether indicating *C. salviifolius* as the most phenolics-rich extract and the extract with the greatest metal reduction capacity. Indeed, the study of plant-based extracts rich in phenolic acids has been reported to strongly and positively correlate with antioxidant activity [45], thus reinforcing the significance of these results in *Cistus* HEs. Moreover, the structural–activity relationship behind this observation results from the fact that a higher abundance of COOH groups augments the radical-scavenging potential of phenolic OH groups, ultimately promoting the antioxidant effect of phenolic acids [46]. In contrast, the HE from *C*. *ladanifer* is apparently more influenced by non-aromatic compounds, such as polysaccharides and aliphatic structures. Indeed, the nature of this spectral profile agrees with the comparatively weaker antioxidant activity observed in *C*. *ladanifer*, particularly in cell-free assays. Furthermore, noticeable differences between *Cistus* spp. HEs were observed in the DPPH^•^ and ABTS^•+^ inhibition assays, which may be the result of the differential scavenging activity demonstrated by phenolics in these HEs upon DPPH^•^ and ABTS^•+^ inhibition [47]. In fact, extracts from *C. salviifolius* have been presenting better antioxidant potential in comparison to other *Cistus* species [48,49].

The predicted cell-free antioxidant activity and the differences observed in the FTIR-ATR spectra of *Cistus* HEs demanded an in-depth analysis of their phenolic composition through HPLC-PDA-ESI-MS^n^. As mentioned before, the three studied *Cistus* species are markedly composed of ellagic acid derivatives in their composition. Structurally, ellagic acid is a dimeric form of gallic acid, exhibiting two lactones, a hydrophobic part made of two hydrocarbon rings and a hydrophilic moiety constituted by four OH groups [4,50]. This phenolic acid is a naturally occurring compound present in numerous medicinal plants and food crops [4], appearing either as complex water-soluble ellagitannins or as ellagic acid itself and derivatives [51]. On the other hand, the presence of flavan-3-ols, such as catechins and gallocatechins, was confirmed in *C. albidus*, despite their absence in *C. ladanifer* and *C. salviifolius*. This is coherent with the work of [13], and assumed to be a chemotaxonomic pattern, since plants from the subgenus *Cistus*, where *C. albidus* belongs, are particularly rich in flavonoids and subgroups like the flavan-3-ols. In comparison, *C. ladanifer* and *C. salviifolius*, both part of the *Leucocistus* subgenus, are mostly rich in ellagic acid derivatives [9], as corroborated by our HPLC-DAD-ESI-MS^n^ analysis. Nevertheless, the work of [8], previously identified flavan-3-ols in methanolic extracts of *C. ladanifer*. Besides flavan-3-ols, *C. albidus* and *C. salviifolius* were characterized by a considerable variety of myricetin and quercetin derivatives, and also some kaempferol-derived glycosides. Altogether, these compounds have been suggested as powerful antioxidants [49], once the higher frequency of OH groups in the B-ring of the aglycone core enhances their antioxidant activity [52]. Also, in the HE from *C. salviifolius*, we quantified an appreciable amount of ligstroside, an iridoid glycoside previously identified in this species [42], which is associated with the antioxidant capacity of several natural matrices like olives [53]. From this phenolic profile, it is conceivable that the observed cell-free antioxidant results found for *C. salviifolius* must be a mixed contribution of flavonol derivatives, ellagic acid derivatives, and ligstroside. On the other hand, *C. ladanifer* extract was shown to be poor in quercetin and myricetin derivatives, in agreement with previous chemical characterizations [38].

Surprisingly, in these *Cistus* spp. HEs, despite the predominance of ellagic acid derivatives like terflavin A and cistusin, other ubiquitously found ellagitannins such as punicalin and punicalagin [9,13,38,54] were not identified. This is possibly due to differences in the used solvents and extraction methodologies, harvesting period, or even edaphoclimatic and geographical influences, relevant to the Côa Valley (Portugal) microclimate from where these plants were harvested. Ellagitannins, classified as hydrolysable tannins, are primarily derived from gallotannins and are by far the largest and most complex family of tannins [55]. These molecules are built from the oxidative C-C coupling between at least two adjacent galloyl units, resulting in the formation of hexahydroxydiphenoyl (HHDP) moieties esterified to a glycosidic residue [51,55]. As such, terflavin A originally identified in the leaves of *Terminallia catttapa* L. (Combretaceae) by Tanaka and collaborators [56], alongside with terflavin B, were firstly hypothesized to be important biosynthetic precursors of punicalin and punicalagin. Further evidence about the biogenesis of these compounds was demonstrated in *Terminallia chebula* Retz. [57]. Meanwhile, the chemistry of cistusin, isolated from the leaves’ extracts of *Cistus × incanus* L. [36], was only recently elucidated as being structurally similar to terflavin A and punicalagin. Bearing in mind the structural similarity and biosynthetic relationships between these ellagitannins, it is conceivable that their properties may align with what is known for compounds like punicalin and punicalagin, or ellagic acid itself. Most of the bioactive potential of these molecules arises from the presence of four OH groups and lactone systems that enable these compounds to counteract both RNS and ROS [50]. While the bioactive potential of punicalagin and punicalin [51] has been widely studied, little is still known about the bioactive potential of ellagitannins like terflavin A and cistusin present in the genus *Cistus* [36].

According to ISO 10993-5:2009 guidelines [58] and OECD recommendations for cytotoxicity assays, concentrations that maintain ≥70% cell viability do not exhibit cytotoxic effects. However, to ensure that the bioactive effects evaluated on cells were not confounded with cytotoxic effects, we deliberately selected concentrations that exhibited no detectable toxicity and that resembled the control conditions. Therefore, from our cytotoxic assessment, *C. ladanifer* was the only extract with no significant deleterious effect on both cells’ metabolic activity and mass. Indeed, the work carried out by Andrade and collaborators [59] found that extracts from *C. ladanifer* promoted metabolic performance in a cell line of skin fibroblasts. In turn, *C. albidus* HE was the most toxic one, possibly given the presence of flavan-3-ols like gallocatechin and (+)-catechin, which were otherwise absent in *C. salviifolius* and *C. ladanifer*. Interestingly, the synergistic effect of catechins has been assumed as a possible mechanism that explains the antiproliferative effect in tumor cell lines [60]. From this perspective, it is plausible to assume that catechins may play a role in the higher toxicity of *C. albidus* in normal skin cells. Lastly, the work of Guzelmeric and collaborators [48] reported that HEs of *C. salviifolius* significantly impaired the growth of both 2D and 3D models of human pancreatic cancer but did not significantly affect the growth of human dermal fibroblasts. Noteworthy, and in line with what is further discussed, the potential of *C. salviifolius* and *C. ladanifer* extracts at their highest non-toxic concentrations (0.4 mg/mL and 1 mg/mL, respectively) was evaluated to ensure their biocompatibility for human skin application. Importantly, both extracts did not induce skin irritation, as demonstrated following OECD Test Guidelines, thereby confirming their safety profile.

Skin fibroblasts are the principal cell type in the dermal layer, having a fundamental role in the production of the extracellular matrix, such as fibronectin, collagen, elastin, and glycosaminoglycans. These are key components that maintain the skin’s elasticity and hydration [61]. However, given the permanent exposure of the skin to environmental hazards like UV radiation and pollutants, an imbalance may result in damaged proteins, lipids, and DNA, which contribute to the development of aging-related stressed skin [4]. As such, the assessment of the antioxidant protective effect of bioactive compounds in skin fibroblasts has been successfully employed by subjecting these cells to in vitro treatment with oxidative stressors like *t*-BHP and H_2_O_2_ [25,43]. From our results, *C. ladanifer* and *C. salviifolius* stood out for their cytoprotective capacity and as cellular oxidative modulators in NHDF cells. As previously discussed, since ellagic acid derivatives were commonly identified as major compounds in both species, they are likely to be responsible for the identified pharmacological activities. As such, an extract from *T. catappa* showed cytoprotective effects and reduced ROS levels by 23.1% in H_2_O_2_-stimulated human skin fibroblasts (Hs68 cell line) [62]. In turn, Dudonné and collaborators [63], using DNA macroarrays in a model of replicative senescence in NHDF cells, demonstrated that plant extracts rich in ellagitannins, such as castalagin and vescalagin, were able to upregulate the expression of catalase, a key antioxidant enzyme involved in the coordinated reduction and neutralization of reactive oxygen species (ROS) alongside other cellular antioxidant defenses. Another investigation proved the cytoprotective effect of the ellagitannin punicalagin in skin fibroblasts affected by UVA radiation by augmenting cells’ viability and reducing ROS levels generated by photooxidative stress [61]. Also related to this, the protective effect of a standardized pomegranate extract rich in ellagitannins was elucidated previously in UVA- and UVB-stimulated SKU-1064 human skin fibroblasts [64].

For instance, the potential anti-inflammatory effect of our extracts was investigated using LPS-stimulated RAW 264.7 macrophages, which is a well-known in vitro model of inflammation. When external LPS binds to the toll-like receptor 4 (TLR4) at the macrophages’ surfaces, it promotes the translocation of the Nuclear factor kappa-light-chain-enhancer of activated B cells (NF-κB) into the nucleus [24,65], inducing the expression of an arsenal of pro-inflammatory mediators and cytokines, including nitric oxide (NO), prostaglandin E2 (PGE2), tumor necrosis factor (TNF-α), IL-1β, and IL-6 [66]. Our results revealed the remarkable ability of *C. ladanifer* HE to inhibit NO production, indicating significant anti-inflammatory potential. In contrast, *C. salviifolius* and *C. albidus* HEs did not exhibit any notable anti-inflammatory activity. Interestingly, the ethanol-based extract of the stem bark of *T. catappa*, essentially composed of ellagic acid derivatives, like terflavin A and castalagin isomers, was demonstrated to reduce IL-1β levels and nitrites production in LPS-stimulated RAW 264.7 macrophages [67]. Another study with this plant species investigated a methanolic extract particularly rich in the ellagitannin α-punicalagin, showing that it reduces the LPS-induced production of NO and other inflammatory markers, besides an evident reduction in ROS levels [68]. Similarly, punicalin and ellagic acid from pomegranate peels were shown to reduce nitrite levels in LPS-activated macrophages, as well as a series of pro-inflammatory cytokines (TNF-α, IL-1β, and IL-6) [69]. Interestingly, the chemical structure of ellagitannins present in pomegranate peels, and the HHDP moieties, flavogallonyl and/or gallagyl parts, were evidenced as fundamental functional groups involved in the inhibition of NO production [70]. Overall, these studies are particularly relevant, reinforcing the assumption that the identified ellagitannins, terflavin A and cistusin, are fundamental for the inhibition of cellular nitrite production promoted by the *C. ladanifer*’s HE herein demonstrated.

Additionally, senescent fibroblast cells are known to exhibit several key hallmarks, from which increased lysosomal β-galactosidase activity stands out [24,71]. According to our work, *C. ladanifer* presents greater potential over *C. salviifolius* and *C. albidus*, and the HE of *C. ladanifer* was tested for cell senescence and shown to counteract the etoposide-induced senescence of NHDFs. Several recent works showed the potential anti-senescence effect of natural products in fibroblasts, such as phenolic-rich extracts of *E. globulus* [24] and *Thymbra capitata* (L.) Cav. [71]. Furthermore, skin aging is not only influenced by ROS and RNS, but also by the dysregulation of multiple enzymatic activities. This enzymatic imbalance contributes to reduced skin moisture, hyperpigmentation, a loss of elasticity, and wrinkle formation [72]. Bearing in mind these aspects, *C. salviifolius* stood out for its enzymatic inhibitory activity, steadily inhibiting all the tested enzymes at rates comparable to those of the positive controls. Alongside this, the HE of *C. ladanifer* also presented a remarkable potential to inhibit elastase. Previous works have suggested that ellagitannins, due to their glucosyl moiety connected to HHDP moieties, interact with the elastase’s aromatic side chains [73]. As discussed before, the extracts content in terflavin A and cistusin are apparently major drivers of the elastase inhibitor role played by HEs of *C. salviifolius* and *C. ladanifer*. In fact, a recent investigation testing extracts from *Cytinus hypocistis* (L.) L. confirmed that hydrolysable tannins like ellagic acid derivatives are responsible for the observed anti-aging properties, namely those related to elastase inhibition [74]. In our work, although *C. ladanifer* HE exhibited comparatively lower activity (~30% inhibition), it was still identified as a potential inhibitor of tyrosinase. On the other hand, *C. albidus* presented promising inhibition potential against tyrosinase and elastase, inhibiting around 50% of both enzymes. The HEs of *C. ladanifer* and *C. albidus* showed no inhibitory potential against hyaluronidase. Based on these cell-free anti-aging findings, *C. salviifolius*, followed by *C. ladanifer*, emerge as the most promising candidates for the development of advanced formulations containing bioactive ingredients that target skin remodeling enzymes.

Based on the provided phenolic profile and compound quantification (mg/g extract), the tested non-toxic extracts’ solutions delivered significantly different amounts of major ellagitannins to cells, which translated into different cytoprotective, antioxidant, and anti-inflammatory potentials. Specifically, *C. ladanifer* HE (1 mg/mL) provides 6.4 µg/mL of terflavin A and 4.7 µg/mL of cistusin. Meanwhile, *C. salviifolius* (0.4 mg/mL) delivers 7.9 µg/mL of terflavin A and 1.7 µg/mL of cistusin. The differences become even more pronounced for *C. albidus* HE, where a 0.2 mg/mL extract solution results in approximately 0.5 µg/mL of both terflavin A and cistusin being administered to cells. It is therefore plausible to hypothesize that these quantitative differences in ellagitannin content per extract solution applied to macrophages are one of the major reasons behind the exclusively anti-inflammatory role of *C. ladanifer* HE, as well as for its antioxidant and cytoprotective activities in NHDFs. The authors point out that although the three studied HEs share similar qualitative profiles, their quantitative differences result in distinct biological inputs at the cellular level. In particular, the higher delivery of ellagitannins by *C. ladanifer* HE, allied to its lower cytotoxicity and combined with its specific phytochemical ratios, may contribute to its stronger and more consistent activity at the cellular level. Conversely, the lower amounts of these key compounds in the *C. albidus* HE may limit its efficacy despite the superficial compositional similarities. These findings underscore the importance of considering not only qualitative composition but also the effective concentrations of individual bioactive constituents that reach cells when interpreting extract bioactivity.

Lastly, the authors recognize the limitations of this study that pave the way for future work. Firstly, the biological activities were evaluated only in vitro, which limits extrapolation to the complexity of human skin. In light if this, confirmation in ex vivo or in vivo models is needed. Secondly, the use of extracts prevented the identification of the specific compounds driving each bioactivity, highlighting the need for the fractionation or targeted isolation of major compounds, specifically the ellagitannins identified. Also, mechanistic pathways underlying the antioxidant, anti-inflammatory, and anti-senescence effects were not explored, and should be addressed in the next step through molecular-level investigations. Finally, broader toxicological and formulation-related assessments are required to substantiate the anti-aging potential for human skin of the HE of *C. ladanifer*, before translational application into the cosmetics industry.

## 5. Conclusions

This study revealed the potential of plants from the genus *Cistus* as candidates for the obtention of natural bioactive compounds targeting skin aging. Among the tested extracts, *C. ladanifer*, notably rich in ellagitannins, emerged as the most promising one. Its potent antioxidant and cytoprotective activity, potential anti-inflammatory effect, and remarkable anti-senescence activity, in combination with significant anti-enzymatic effects, underscore its suitability as a candidate to include in anti-aging cosmetics. Importantly, the absence of skin irritation in OECD-compliant tests supports the safety profile of such natural extracts, namely *C. ladanifer*, for future topical applications. By bridging modern phytochemical and pharmacological evidence, these findings offer direct relevance to the industry, particularly in the context of growing consumer demand for natural, eco-conscious, and scientifically substantiated skincare solutions. Lastly, the HE from *C. ladanifer* is highlighted as a promising candidate for inclusion in innovative skin anti-aging formulations in the cosmetics industry. 

## Figures and Tables

**Table 1:** FTIR-ATR wavenumbers and functional groups’ assignments for the HEs in this study.

Region (cm^−1^)	Assignment	References	Ca	Cl	Cs
*a* 3368–3326	*ν*(O–H)	[27,28]	3326	3326	3368
*b* 2938–2931	*ν*_antisym_(CH_3_ and CH_2_), aliphatic compounds	[29,30]	2935	2931	2938
*c* 2895–2882	*ν*_sym_(CH_3_ and CH_2_), aliphatic compounds	[31,32]	2882	2886	2895
*d* 1707–1693	*ν*(C=O) in COOH	[29,30,33]	1693	1707	1701
*e* 1612–1608	*ν*(C=C) aromatic ring	[27,29,33]	1608	1609	1612
*f* 1514	*ν*(C–C) aromatic ring	[27,28,31]	1514	1514	1514
*g* 1449–1444	*δ*(CH_3_ and CH_2_), aliphatic compounds; polysaccharides; *ν*(C–C) aromatic ring	[28,29,30]	1446	1444	1449
*h* 1413–1410	*δ*(C–H)	[29,33,34]	1410	1413	1412
*i* 1355–1346	*δ*(CH_2_), *ν*(C–C) polysaccharides (pectin); *δ*(C–OH)	[28,29,30,32]	1346	1355	1353
*j* 1261–1230	*δ*(C–H), *ν*(C–OH)	[27,31]	1261sh; 1237	1261sh; 1230	1261sh; 1230
*k* 1145–1102	*ν*(C–O–C) ester, *ν*(C–O) and *δ*(C–OH) carbohydrates/non-aromatics	[29,30]	1141; 1102	1145; 1102	1145; 1102
*l* 1063–1051	*ν*(C–O) and *ν*(C–C) carbohydrates/non-aromatics	[30,32]	1051	1054	1063

Abbreviations: antisym, antisymmetric; sym, symmetric; *ν*, stretching; *δ*, bending; sh, shoulder; Ca, *C. albidus*; Cl, *C. ladanifer*; and Cs, *C. salviifolius*.

**Table 2:** Peaks, retention time (Rt) in min, wavelengths (λ_max_) in nm, mass spectral data ([M-H]^−^ and MS^n^ (*m*/*z*)), tentative identification, and quantification (mg g^−1^ extract) of phenolic compounds in the HE of *C. albidus*, *C. ladanifer*, and *C. salviifolius*. Major compounds identified are highlighted in bold.

***C. albidus***							
**Peak**	**Rt**	***λ*** **_max_**	**[M-H]^−^**	**MS^n^**	**Tentative Identification**	**Quantification**	**References**
**1*^Ca^***	**4.87**	**271**	**305**	**MS^2^: 179(100), 165(15)**	**Gallocatechin**	**3.46 ± 0.03**	[35]
**2*^Ca^***	**5.76**	**262**	**1085**	**MS^2^: 542(100)**	**Terflavin A**	**2.50 ± 0.10**	[36]
3*^Ca^*	6.55	309	337	MS^2^: 191(100), 173(12), 163(5)	3-*O*-*p*-coumaroylquinic acid	1.13 ± 0.06	[37]
**4*^Ca^***	**6.82**	**260**	**1251**	**MS^2^: 625(45), 603(10)**	**Cistusin**	**2.43 ± 0.10**	[36]
5*^Ca^*	7.50	280	289	MS^2^: 245(100), 203(14)	(+)-Catechin	1.21 ± 0.06	[36]
6*^Ca^*	15.39	357	479	MS^2^: 317(100)	Myricetin-*O*-hexoside isomer I	1.12 ± 0.01	DAD/MS
7*^Ca^*	15.62	357	479	MS^2^: 317(100)	Myricetin-*O*-hexoside isomer II	0.93 ± 0.03	DAD/MS
8*^Ca^*	17.73	358	563	MS^2^: 521; MS^3^: 479(35), 317(85), 316(100)	Myricetin 3-*O*-(6″-malonyl)hexoside	1.20 ± 0.03	[35]
9*^Ca^*	17.79	358	449	MS^2^: 317(100)	Myricetin-*O*-pentoside	1.01 ± 0.01	[35]
**10*^Ca^***	**17.90**	**357**	**463**	**MS^2^: 317(100)**	**Myricetin-*O*-deoxyhexoside**	**5.29 ± 0.04**	[35]
11*^Ca^*	18.84	355	463	MS^2^: 301(100)	Quercetin-*O*-hexoside	0.89 ± 0.01	[35]
12*^Ca^*	20.83	357	549	MS^2^: 505(100); MS^3^: 301(100)	Quercetin 3-*O*-(6″-malonyl)hexoside	1.04 ± 0.05	[35]
13*^Ca^*	21.64	357	433	MS^2^: 301(100)	Quercetin-*O*-pentoside	1.03 ± 0.03	[35]
14*^Ca^*	22.34	354	771	MS^2^: 625(100), 317(32)	Myricetin-*O*-deoxyhexosyl-hexosyl-deoxyhexoside	0.83 ± 0.02	DAD/MS
**15*^Ca^***	**22.78**	**351**	**447**	**MS^2^: 301(100)**	**Quercetin-*O*-deoxyhexoside**	**4.49 ± 0.01**	[35]
16*^Ca^*	33.21	311/347	593	MS^2^: 307(5), 285(100)	kaempferol 7-*O*-(6″-*p*-coumaroyl)hexoside	0.86 ± 0.00	[35]
17*^Ca^*	35.17	288	271	MS^2^: 177(45), 151(100)	Naringenin	0.32 ± 0.00	Standard
18*^Ca^*	37.84	295	329	MS^2^: 167(100), 152(10)	Vanilic acid hexoside	0.08 ± 0.00	[35]
					**Total phenolic acids**	1.21 ± 0.061 ^a^	
					**Total ellagic acid derivatives**	4.92 ± 0.004 ^b^	
					**Total flavan-3-ol**	4.68 ± 0.022 ^b^	
					**Total isoflavonoids**	0.32 ± 0.004 ^c^	
					**Total flavonoids**	18.68 ± 0.13 ^d^	
					**Total phenolic compounds**	29.80 ± 0.04 ^e^	
***C. ladanifer***							
**Peak**	**Rt**	***λ*** **_max_**	**[M-H]^−^**	**MS^n^**	**Tentative Identification**	**Quantification**	**References**
**2*^Cl^***	**5.76**	**262**	**1085**	**MS^2^: 542(100)**	**Terflavin A**	**6.35 ± 0.12**	[36]
**4*^Cl^***	**6.82**	**260**	**1251**	**MS^2^: 625(45), 603(10)**	**Cistusin**	**4.77 ± 0.20**	[36]
5′*^Cl^*	10.04	356	593	MS^2^: 473(54), 383(38), 353(65)	Apigenin-*C*-dihexoside	1.08 ± 0.06	[38]
5″*^Cl^*	11.70	344	373	MS^2^: 358(34), 343(100), 328(12)	Dihydroxy-tetramethoxyflavone	0.67 ± 0.04	[39]
5‴*^Cl^*	11.92	277	327	MS^2^:165(100), 101(10)	3.4′-Dihydroxypropiophenone-3-glucoside	1.83 ± 0.02	[39]
5⁗*^Cl^*	13.74	346	625	MS^2^: 463(100), 301(13)	Quercetin-*O*-hexoside-hexoside	0.80 ± 0.03	DAD/MS
6′*^Cl^*	14.65	345	609	MS^2^:301(100)	Quercetin-deoxyhexosyl-hexoside	0.53 ± 0.02	DAD/MS
6″*^Cl^*	15.90	278	507	MS^2^: 463(27), 313(100)	Unknown	n.d.	[40]
6‴*^Cl^*	16.20	351	449	MS^2^: 269(100), 251(54)	Dihydrosinapoyl conjugate	0.10 ± 0.01	[41]
18′*^Cl^*	39.05	350	299	MS^2^: 285(100), 255(62), 227(28)	Kaempferol methylether	0.64 ± 0.02	[38]
					**Total phenolic acids**	0.10 ± 0.01 ^a^	
					**Total ellagic acid derivatives**	11.12 ± 0.09 ^b^	
					**Total flavonoids**	5.55 ± 0.18 ^c^	
					**Total phenolic compounds**	16.77 ± 0.27 ^d^	
***C. salviifolius***							
**Peak**	**Rt**	***λ*** **_max_**	**[M-H]^−^**	**MS^n^**	**Tentative Identification**	**Quantification**	**References**
**1*^Cs^***	**5.76**	**262**	**1085**	**MS^2^: 542(100)**	**Terflavin A**	**19.75 ± 0.35**	[36]
**2*^Cs^***	**6.82**	**260**	**1251**	**MS^2^: 625(45), 603(10)**	**Cistusin**	**4.25 ± 0.13**	[36]
**3*^Cs^***	**9.93**	**272**	**453**	**MS^2^: 313(100), 169(12), 151(5)**	**Ligstroside derivative**	**13.92 ± 0.32**	[42]
4*^Cs^*	14.09	356	631	MS^2^: 479(100), 317(54)	Myricetin-galactosyl-hexoside	0.86 ± 0.04	DAD/MS
5*^Cs^*	15.39	357	479	MS^2^: 317(100)	Myricetin-*O*-hexoside isomer I	1.79 ± 0.01	DAD/MS
6*^Cs^*	15.62	357	479	MS^2^: 317(100)	Myricetin-*O*-hexoside isomer II	1.03 ± 0.04	DAD/MS
7*^Cs^*	17.27	354	615	MS^2^: 463(100), 301(45)	Quercetin-galactosyl-hexoside	0.73 ± 0.01	DAD/MS
8*^Cs^*	17.79	358	449	MS^2^: 317(100)	Myricetin-*O*-pentoside	1.60 ± 0.07	[35]
9*^Cs^*	17.90	357	463	MS^2^: 317(100)	Myricetin-*O*-deoxyhexoside	1.88 ± 0.04	[35]
10*^Cs^*	18.84	355	463	MS^2^: 301(100)	Quercetin-*O*-hexoside	1.26 ± 0.05	[35]
11*^Cs^*	20.83	357	549	MS^2^: 505(100); MS^3^: 301(100)	Quercetin 3-*O*-(6″-malonyl)hexoside	0.70 ± 0.01	[35]
12*^Cs^*	21.64	357	433	MS^2^: 301(100)	Quercetin-*O*-pentoside isomer I	1.88 ± 0.03	[35]
13*^Cs^*	21.83	357	433	MS^2^: 301(100)	Quercetin-*O*-pentoside isomer II	2.63 ± 0.01	[35]
14*^Cs^*	22.34	354	771	MS^2^: 625(100), 317(32)	Myricetin-*O*-deoxyhexosyl-hexosyl-deoxyhexoside	0.75 ± 0.01	DAD/MS
15*^Cs^*	33.21	311/347	593	MS^2^: 307(5), 285(100)	Kaempferol 7-*O*-(6″-*p*-coumaroyl)hexoside	1.07 ± 0.04	[35]
					**Total ellagic acid derivatives**	24.00 ± 0.22 ^a^	
					**Total flavonoids**	15.74 ± 0.38 ^b^	
					**Total secoiridoid glycosides**	13.92 ± 0.32 ^b^	
					**Total phenolic compounds**	54.08 ± 0.75 ^c^	

Values represent the mean ± standard deviation. The statistical analysis was carried out by one-way ANOVA, followed by Tukey’s post hoc test (*p* < 0.05), regarding major groups of compounds (e.g., total phenolic acids) identified in each HE. Significant differences are represented by superscript letters (a–e). Rt: Retention time in min; λ_max_: wavelength (nm) of maximum absorption in the UV–visible region; [M-H]^−^: deprotonated ion (negative ion mode) (*m*/*z*); MS^n^ fragment ions generated in MS^2^ and/or MS^3^ spectra (*m*/*z*) and relative abundance in brackets (% base peak).

**Table 3:** Cell-free effect of HEs on the inhibition (%) of enzymes involved in skin aging.

	Hyaluronidase	Tyrosinase	Elastase
Ca (0.2 mg/mL)	n.a.	53.53 ± 8.71 ****	53.50 ± 5.59 *
Cl (1.0 mg/mL)	n.a.	27.75 ± 4.10 ****	64.16 ± 8.18
Cs (0.4 mg/mL)	94.11 ± 7.98	96.80 ± 2.45	75.72 ± 8.82
Positive control ^a^	98.76 ± 8.20	93.31 ± 5.54	68.17 ± 5.51

^a^ EGCG at 200 µM, KA at 800 µM, and EGCG at 250 µM were used as positive controls for the hyaluronidase, tyrosinase, and elastase assays, respectively. Values express % of enzyme inhibition and represent the mean ± SD of at least five independent experiments performed in triplicate. The statistical analysis was carried out by one-way ANOVA followed by Dunnett’s multiple comparison test (* *p* < 0.05 and **** *p* < 0.0001 versus positive control). Abbreviations: n.a., not active; EGCG, Epigallocatechin gallate; KA, Kojic acid; Ca, *C. albidus*; Cl, *C. ladanifer*; Cs, *C. salviifolius*.

## Data Availability

The original contributions presented in this study are included in the article and Supplementary Materials. The raw data supporting the conclusions of this article will be made available by the authors on request.

## Supplementary Materials

The following supporting information can be downloaded at: https://www.mdpi.com/article/10.3390/antiox15010149/s1, Table S1. Plant material harvested in Côa Valley (Portugal) assessed in this study. Information includes taxa (plant species and botanical family), the collector’s name, herbarium voucher specimen code that is deposited at the Herbarium of the University of Aveiro (AVE), date and detailed harvesting site, the employed extraction method, the plant’s parts used for extraction, and respective yield of extraction values (%) presented as the mean ± standard deviation of three independent experiments; Table S2. Total phenolic content (TPC, mg GAE g^−1^ extract DW), total flavonoid content (TFC, mg QE g^−1^ extract DW), cupric (CUPRAC) and ferric (FRAP) reducing powers (mg TE g^−1^ extract DW), and free radical scavenging activity (DPPH and ABTS) presented as IC_50_ values (mg/mL); Figure S1. FTIR-ATR spectra in the range 1800–800 cm^−1^ of the standard phenolic compounds gallic acid (A), myricetin (B), quercetin (C), catechin (D), kaempferol (E), epicatechin (F), ferulic acid (G), and *p*-coumaric acid (H); Figure S2. Illustrative phenolic profiles of the HE of *C. salviifolius* (A,B), *C. albidus* (C,D), *C. ladanifer* (E,F) recorded at 280 and 370 nm, respectively. Abbreviations: mAU, milli-absorbance unit; Cs, *C. salviifolius*; Ca, *C. albidus*; Cl., *C. ladanifer*; Figure S3. Effect of the HE of *C. albidus* (A_1_,A_2_), *C. ladanifer* (B_1_,B_2_), *C. salviifolius* (C_1_,C_2_) on the metabolic activity and cell mass of Raw 264.7 macrophages. Cells were treated with HE (0.2–1.0 mg/mL) and LPS (0.1 µg/mL) for 24 h, and metabolic activity effects were evaluated through the Alamar blue^®^ and SRB assays. Untreated cells were used as the control (CTRL). The results are expressed as percentage (%) of metabolic activity and cell mass relative to the CTRL and represent the mean ± SD of four independent experiments, each one performed in triplicate. The statistical analysis was carried out by one-way ANOVA followed by Dunnett’s multiple comparison test (* *p* < 0.05, ** *p* < 0.01, *** *p* < 0.001, and **** *p* < 0.0001 versus CTRL).

## Abbreviations

The following abbreviations are used in this manuscript:

ABTS^•+^	2,2′-azino-bis(3-ethylbenzothiazoline-6-sulfonic acid) radical cation
ANOVA	Analysis of variance
AVE	Herbarium of the University of Aveiro
BHT	Butylated hydroxytoluene
CUPRAC	Cupric ion reducing antioxidant capacity
Ca	*C. albidus*
Cl	*C. ladanifer*
Cs	*C. salviifolius*
DMEM	Dulbecco’s modified Eagle’s medium
DPPH**^•^**	2,2-Diphenyl-1-picrylhydrazyl radical
DW	Dry weight
EGCG	(-)Epigallocatechin Gallate
Eto	Etoposide
FBS	Fetal bovine serum
FRAP	Ferric reducing antioxidant power
GAE	Gallic acid equivalents
FTIR-ATR	Fourier-transformed infrared spectroscopy (FTIR) in attenuated total reflectance (ATR) mode
HaCat	Immortalized human keratinocytes cell line
HE	Hydroethanolic extract (80:20%, *v*/*v*) (EtOH 80%)
HPLC-DAD-ESI/MS^n^	High-performance liquid chromatography coupled to photodiode array detection and electrospray ionization tandem mass spectrometry
H_2_DCFDA	2′,7′-Dichlorodihydrofluorescein diacetate
IL	Interleukin
KA	Kojic acid
LC	Liquid chromatography
LPS	Lipopolysaccharide
min	Minutes
MS	Mass spectrometer
MTT	Thiazolyl blue tetrazolium bromide
NF-κB	Nuclear factor kappa-light-chain-enhancer of activated B cells
NHDFs	Normal human dermal fibroblasts cell line
NO	Nitric oxide
Nrf2	Nuclear factor erythroid 2-related factor 2
OECD	Organization for Economic Co-operation and Development
OH	Hydroxyl group
PCA	Principal component analysis
QE	Quercetin equivalents
RHE	Reconstructed human epidermis
RNS	Reactive nitrogen species
ROS	Reactive oxygen species
RT	Room temperature
Rt	Retention time
SD	Standard deviation
SDS	Sodium dodecyl sulfate
Sec	Seconds
SRB	Sulforhodamine B
TE	Trolox equivalents
TFC	Total flavonoid content
TLR-4	Toll-like receptor 4
TNF-α	Tumor necrosis factor α
TPC	Total phenolic content
UV	Ultraviolet
X-gal	X-galactose
