# Supplementary material for: Targeting Skin Aging Hallmarks In Vitro: Antioxidant, Anti-Inflammatory, and Anti-Senescence Effects of Phenolic-Rich Extracts from Cistus L. Species
Source: Antioxidants (Basel). 2026 Jan 22;15(1):149. doi: 10.3390/antiox15010149 (PMC12838026; doi:10.3390/antiox15010149)
Supplement: Supplementary file 1 [file antioxidants-15-00149-s001.zip › antioxidants-4029619-supplementary.pdf]

Supplementary Materials

Table S1. Plant material harvested in Côa Valley (Portugal) assessed in this study. Information includes taxa (plant species and botanical family), the collector's name, herbarium voucher specimen code that is deposited at the Herbarium of the University of Aveiro (AVE), date and detailed harvesting site, the employed extraction method, the plant's parts used for extraction, and respective yield of extraction values (%) presented as the mean ± standard deviation of three independent experiments.

Taxa	**Collector**	**Herbarium voucher specimen**	**Date of harvesting**	**Harvesting site**	**Type of extraction**	**Plant parts used for extraction**	**Extraction yield (%)**
Cistus albidus L. (Ca) (Cistaceae)	Mário Pedro Marques	AVE127	April 2022	Old train station of V. N. de Foz Coa (41°05'01.7"N 7°06'18.6"W)	Ethanol-water (80:20, v/v)	Flowering aerial parts	13.911 ± 0.757
Cistus ladanifer L. subsp. *ladanifer* (Cl) (Cistaceae)	Mário Pedro Marques	AVE230	March 2022	Côa Parque Foundation, V. N. de Foz Coa (41°04'49.7"N 7°06'41.0"W)	Ethanol-water (80:20, v/v)	Flowering aerial parts	9.347 ± 0.677
Cistus salviifolius L. (Cs) (Cistaceae)	Mário Pedro Marques	AVE120	April 2022	Côa Parque Foundation, V. N. de Foz Coa (41°04'49.7"N 7°06'41.0"W)	Ethanol-water (80:20, v/v)	Flowering aerial parts	16.212 ± 2.274

Table S2. Total phenolic content (TPC, mg GAE g^{-1} extract DW), total flavonoid content (TFC, mg QE g^{-1} extract DW), cupric (CUPRAC) and ferric (FRAP) reducing powers (mg TE g^{-1} extract DW), and free radical scavenging activity (DPPH and ABTS) presented as IC_{50} values (mg/mL).

**Samples**	**TPC**	**TFC**	**CUPRAC**	**FRAP**	**DPPH**	**ABTS**
**Cl**	55.498 ± 12.598^{a}	5.517 ± 0.071^{a}	89.102 ± 10.88^{a}	119.581 ± 8.399^{a}	0.15 ± 0.019 ^{a}	0.893 ± 0.048^{a}
**Ca**	136.964 ± 18.741^{b}	39.505 ± 3.804^{b}	378.523 ± 27.612^{b}	175.624 ± 5.999^{b}	0.045 ± 0.003^{b}	0.265 ± 0.037^{b}
**Cs**	244.559 ± 24.449^{c}	46.351 ± 2.323^{c}	678.517 ± 65.08^{c}	422.618 ± 5.026^{c}	0.074 ± 0.006 ^{b}	0.19 ± 0.006^{b,c}
**BHT**	-	-	-	-	0.123 ± 0.018 ^{a,c}	0.164 ± 0.025^{c,d}

Abbreviations: Cl. *C. ladanifer*; Ca, *C. albidus*; Cs, *C. salviifolius*; BHT, butylated hydroxytoluene (positive control); DW, Dry weight; GAE, gallic acid equivalents; n.d., not determined; QE, quercetin equivalents. Values represent the mean ± standard deviation of three independent experiments performed in triplicates. For each column, different superscript letters (a-d) indicate significant differences. The statistical analysis was carried out by one-way analysis of variance (ANOVA), followed by Tukey's post hoc test ($p<0.05$) for comparisons between three or more groups.

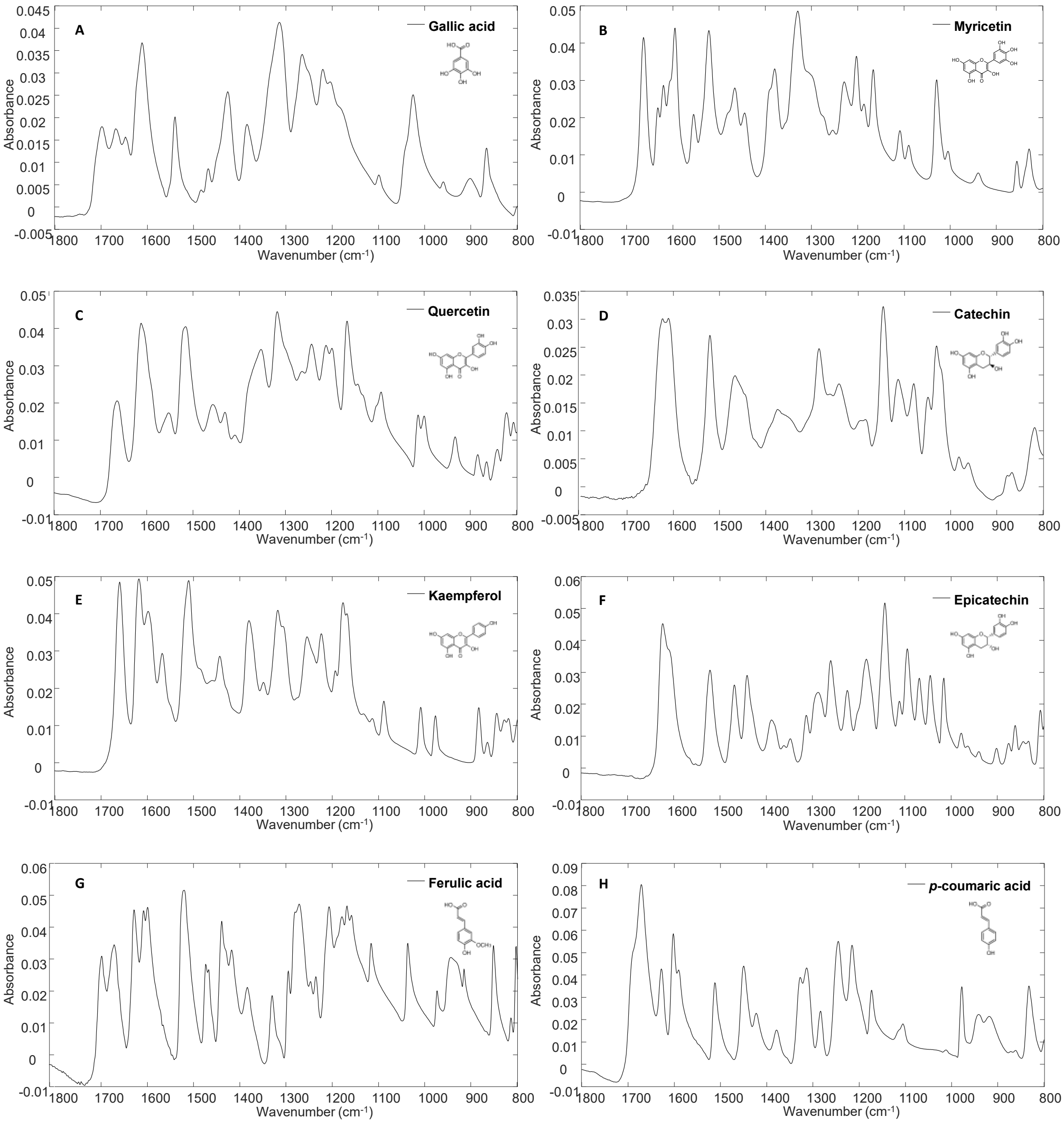

Figure S1. FTIR-ATR spectra in the range 1800 – 800 cm^{-1} of the standard phenolic compounds gallic acid (A), myricetin (B), quercetin (C), catechin (D), kaempferol (E), epicatechin (F), ferulic acid (G), and *p*-coumaric acid (H).

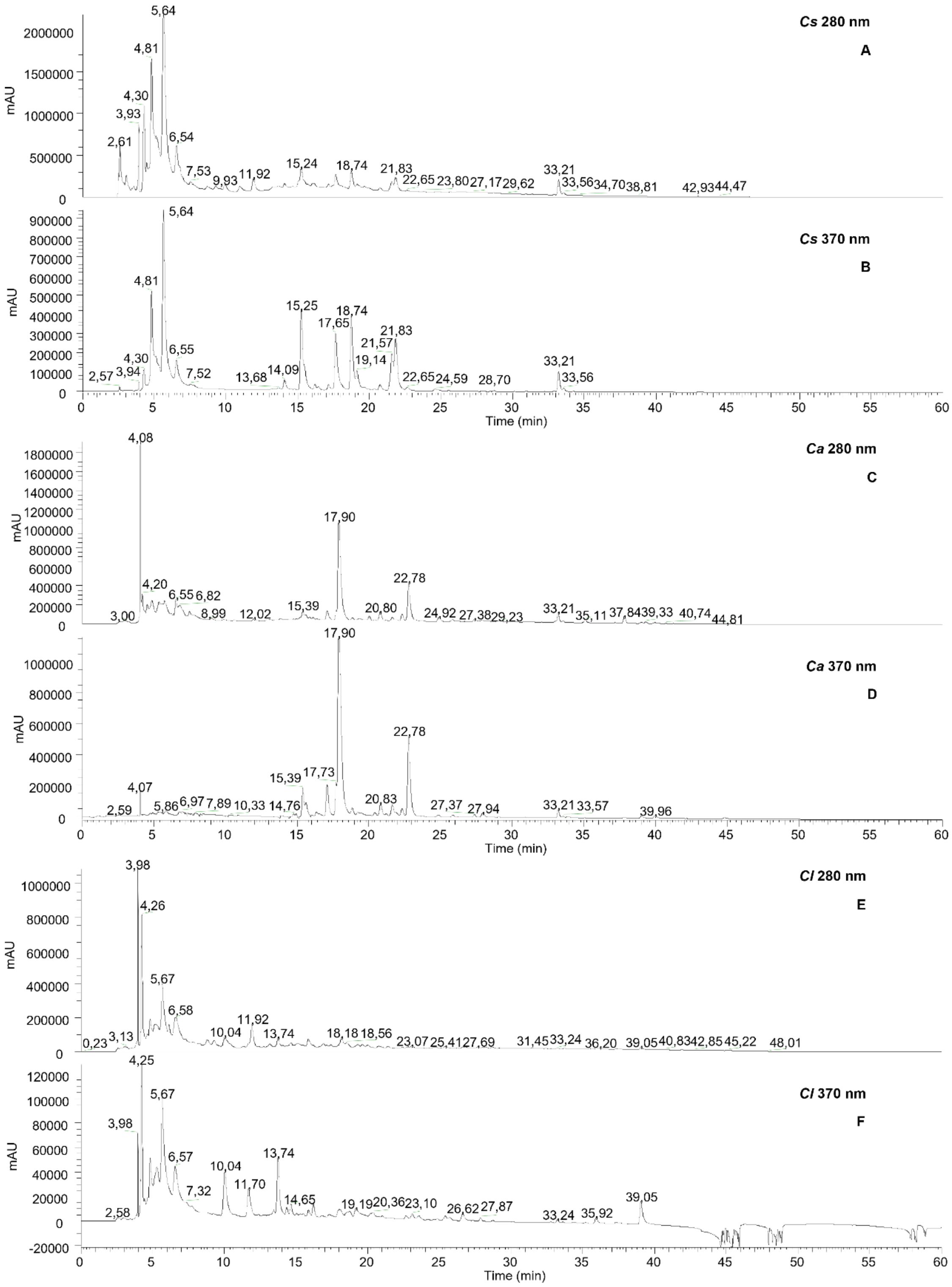

Figure S2. Illustrative phenolic profiles of the HE of *C. salviifolius* (A and B), *C. albidus* (C and D), *C. ladanifer* (E and F) recorded at 280 and 370 nm, respectively. **Abbreviations:** mAU, milli-absorbance unit; Cs, *C. salviifolius*; Ca, *C. albidus*; Cl., *C. ladanifer*.

List of standard calibration curves used for quantification of the identified compounds in the following:

***C. albidus* (*Ca*):** Ellagic acid ($y = 26.719x - 317.255$, $R^2 = 0.999$; LOD = 41.20 µg/mL; LOQ = 124.84 µg/mL; peaks 2^{Ca} and 4^{Ca}), (+)-catechin ($y = 84.950x - 23.200$, $R^2 = 0.999$; LOD = 0.17 µg/mL; LOQ = 0.68 µg/mL; peak 1^{Ca} and 5^{Ca}), *p*-coumaric acid ($y = 301.950x + 6966.7$, $R^2 = 0.999$; LOD = 0.68 µg/mL; LOQ = 1.61 µg/mL; peak 3^{Ca}), myricetin ($y = 23.287x - 581.708$, $R^2 = 0.999$; LOD = 61.21 µg/mL; LOQ = 185.49 µg/mL; peaks 6^{Ca}, 7^{Ca}, 8^{Ca}, 9^{Ca}, 10^{Ca}, and 14^{Ca}), quercetin-3-*O*-glucoside ($y = 34.843x - 160.173$, $R^2 = 0.991$; LOD = 0.21 µg/mL; LOQ = 0.71 µg/mL; peaks 11^{Ca}, 12^{Ca}, 13^{Ca}, 15^{Ca} and 16^{Ca}), naringenin ($y = 184.33x + 78.903$, $R^2 = 0.999$; LOD = 0.17 µg/mL; LOQ = 0.81 µg/mL; peak 17^{Ca}), vanillic acid ($y = 29.751x - 28.661$, $R^2 = 0.999$; LOD = 16.65 µg/mL; LOQ = 50.45 µg/mL; peak 18^{Ca}).

***C. ladanifer* (*Cl*):** Ellagic acid ($y = 26.719x - 317.255$, $R^2 = 0.999$; LOD = 41.20 µg/mL; LOQ = 124.84 µg/mL; peaks 2^{Cl} and 4^{Cl}), apigenine-6-*C*-glucoside ($y = 107.025x + 61.531$, $R^2 = 0.999$; LOD = 0.19 µg/mL; LOQ = 0.63 µg/mL; peak $5'^{Cl}$), quercetin-3-*O*-glucoside ($y = 34.843x - 160.173$, $R^2 = 0.991$; LOD = 0.21 µg/mL; LOQ = 0.71 µg/mL; peaks $5''^{Cl}$, $5'''^{Cl}$, $5''''^{Cl}$, $6'^{Cl}$ and $18'^{Cl}$) and sinapic acid ($y = 197.337x + 30.036$, $R^2 = 0.999$; LOD = 1.91 µg/mL; LOQ = 6.01 µg/mL peak $6'''^{Cl}$).

***C. salviifolius* (*Cs*):** Ellagic acid ($y = 26.719x - 317.255$, $R^2 = 0.999$; LOD = 41.20 µg/mL; LOQ = 124.84 µg/mL; peaks 1^{Cs} and 2^{Cs}), oleuropein ($y = 32.226x + 12.416$, $R^2 = 0.999$; LOD = 0.69µg/mL; LOQ = 1.96 µg/mL; peak 3^{Cs}), myricetin ($y = 23.287x - 581.708$, $R^2 = 0.999$; LOD = 61.21 µg/mL; LOQ = 185.49 µg/mL; peaks 4^{Cs}, 5^{Cs}, 6^{Cs}, 8^{Cs}, 9^{Cs}, and 14^{Cs}) and quercetin-3-*O*-glucoside ($y = 34.843x - 160.173$, $R^2 = 0.991$; LOD = 0.21 µg/mL; LOQ = 0.71 µg/mL; peaks 7^{Cs}, 10^{Cs}, 11^{Cs}, 12^{Cs}, 13^{Cs} and 15^{Cs}).

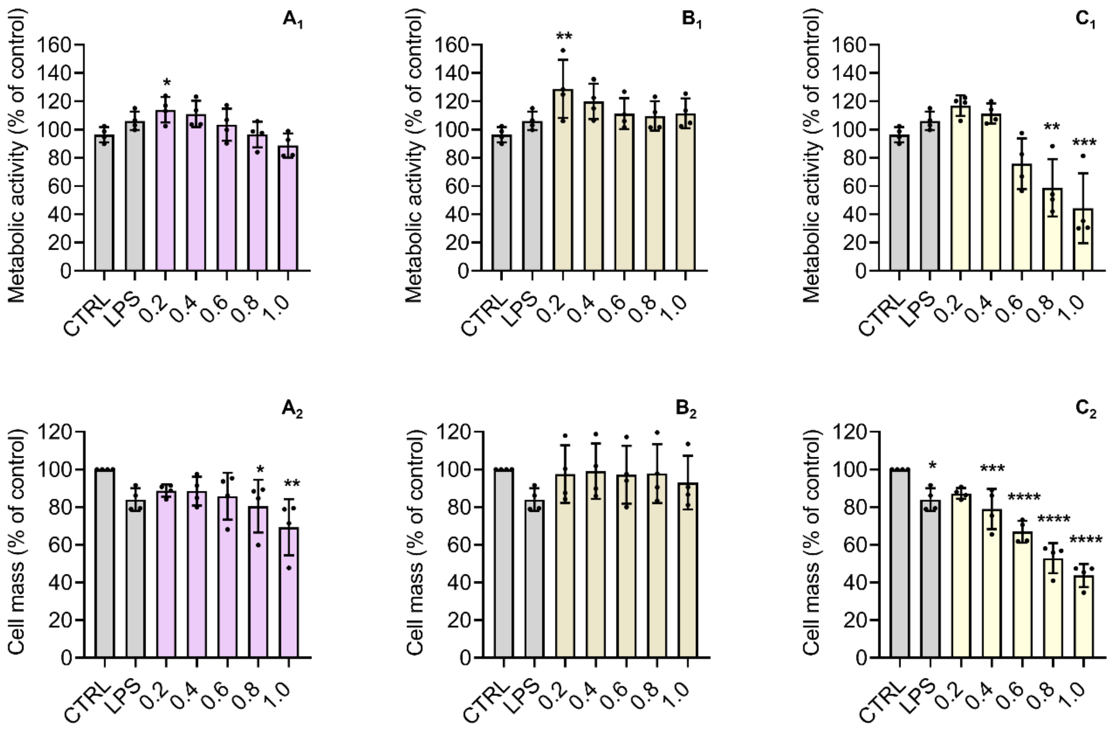

Figure S3. Effect of the HE of *C. albidus* (A_1 and A_2), *C. ladanifer* (B_1 and B_2), *C. salviifolius* (C_1 and C_2) on the metabolic activity and cell mass of Raw 264.7 macrophages. Cells were treated with HE (0.2 – 1.0 mg/mL) and LPS (0.1 µg/mL) for 24 h, and metabolic activity effects were evaluated through the Alamar blue® and SRB assays. Untreated cells were used as the control (CTRL). The results are expressed as percentage (%) of metabolic activity and cell mass relative to the CTRL and represent the mean ± SD of four independent experiments, each one performed in triplicate. The statistical analysis was carried out by one-way ANOVA followed by Dunnett's multiple comparison test (*$p<0.05$, **$p<0.01$, ***$p<0.001$, and ****$p<0.0001$ versus CTRL).
